# A bioinformatics pipeline to search functional motifs within whole-proteome data: a case study of poxviruses

**DOI:** 10.1007/s11262-016-1416-9

**Published:** 2016-12-20

**Authors:** Haitham Sobhy

**Affiliations:** Department of Molecular Biology, Umeå University, 901 87 Umeå, Sweden

**Keywords:** Protein domain, Protein function, Protein annotation, Functional genomics, Comparative genomics, Low-complexity regions (LCRs)

## Abstract

Proteins harbor domains or short linear motifs, which facilitate their functions and interactions. Finding functional motifs in protein sequences could predict the putative cellular roles or characteristics of hypothetical proteins. In this study, we present Shetti-Motif, which is an interactive tool to (i) map UniProt and PROSITE flat files, (ii) search for multiple pre-defined consensus patterns or experimentally validated functional motifs in large datasets protein sequences (proteome-wide), (iii) search for motifs containing repeated residues (low-complexity regions, e.g., Leu-, SR-, PEST-rich motifs, etc.). As proof of principle, using this comparative proteomics pipeline, eleven proteomes encoded by member of Poxviridae family were searched against about 100 experimentally validated functional motifs. The closely related viruses and viruses infect the same host cells (e.g. vaccinia and variola viruses) show similar motif-containing proteins profile. The motifs encoded by these viruses are correlated, which explains why poxviruses are able to interact with wide range of host cells. In conclusion, this in silico analysis is useful to establish a dataset(s) or potential proteins for further investigation or compare between species.

**Electronic supplementary material:**

The online version of this article (doi:10.1007/s11262-016-1416-9) contains supplementary material, which is available to authorized users.

## Introduction

Protein functions and interactions are facilitated by amino acid (aa) sequences, so-called functional motifs, or domains, which participate in various processes, including protein interactions, trafficking, pre- or post-translational regulation, or recruiting enzyme [1–5]. They are either short linear motifs (SLiM), 3–11 residues (e.g., RGD), or long domain, >30 residues (e.g., Zinc finger, ankyrin or tetratricopeptide repeats (TPR)). Motifs may contain repeated residue(s) or region(s) (e.g., L-, SR-, AR- or PEST-rich motifs). Number of databases were established to catalogue these motifs, including PROSITE, ELM, and Minimotif Miner (MnM) databases [6–8]. MnM, MEME Suite, QSLiMFinder, SLiMSearch, 3of5, MotifHound, and DoReMi tools can be used to predict motif(s), pattern(s), or shared consensus within input sequence(s) [9–14]. Another approach uses hidden Markov model (phylo-HMM) to search for evolutionarily conserved functional motifs [15]. These tools were previously reviewed in [13, 16]. Briefly, they offer arena for searching and parsing de novo or pre-defined motifs. They may require sequence alignment, uploading background sequences, or connection to third-party tools or databases. Statistics, based on background sequences to overcome false-positive results, were provided. On the other hand, for finding sequences enriched with residues, EMBOSS provides a tool for finding PEST-rich motif within a query sequence (http://emboss.sourceforge.net/), whereas LCR-eXXXplorer is developed to visualize low-complexity regions (LCRs) [17].

**Fig. 1:**
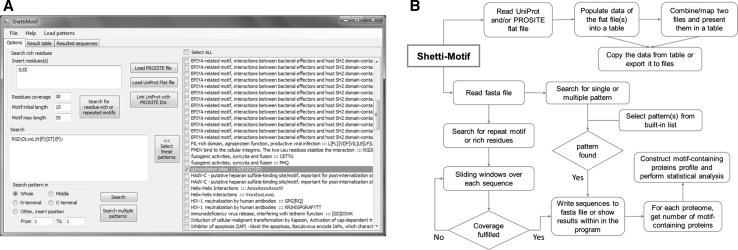
Screenshot of Shetti-Motif main window (**a**), and flowchart of features and method used in this study (**b**)

Shetti-Motif was developed to help experimental biologists to mine for multiple (pre-defined or experimentally validated) motifs, consensus patterns, or motifs enriched with residues within a large dataset of protein sequences (e.g., entire proteome). The tool is interactive, versatile, and user-friendly, Fig. 1. It visualizes UniProt and PROSITE flat files and maps them in a human-readable table.

## Method

Shetti-Motif is standalone and portable program, which is developed in C#.NET. The tool is free for academic uses. The main purpose of the tool is to mine for data within large dataset of sequences, and present them in a human-readable table. The input file is FASTA sequences, UniProt or PROSITE flat files, which are publically available in the databases. All the sequences were downloaded from UniProt, GeneBank and PROSITE (prosite.expasy.org/) websites during October 2015. Three modules were implemented in Shetti-Motif tool.

The first module is searching for x-rich motifs (i.e., motifs enriched with a residue(s), where x is any residue, e.g., Leu-, SR- or PEST-rich motifs) in multiple sequences (entire proteome). Coverage of the residue(s) within motif is the criterion to select the motif. The default coverage value is 30% (e.g., if the length of P-rich motif is 10 aa, P is enriched >3 aa) and can be modified by users. Using sliding window, Shetti-Motif slides over the sequence until residue coverage and motif length thresholds are fulfilled. The tool reports proteins enriched with the input residues, protein length, number of motifs in each protein, motif length, and coverage (number) of residue(s), Figs. S1, S2.

 Shetti-Motif has additional interactive feature, which enables searching for one or multiple consensus pattern among multiple protein sequences, Figs. S3–S6 [18]. Shetti-Motif provides two built-in databases; the first obtained from PROSITE database, while the second obtained from literature, which are validated experimentally, Fig. S3, Tables 1, S1–S3. Users may select patterns from the list, or third-party motif/pattern of interest. Notably, the tool accepts PROSITE pattern syntax, Table S1. The tool uses perfect (exact) text-search method, including regular expression, to search for patterns. By this option, large datasets of proteomes can be parsed efficiently. The outputs are presented in a table or exported to text file, Figs S4–S6. Protein names, number of proteins, and enrichment of the proteins to total number of proteins on the dataset are reported.

**Table 1:** The motif-containing proteins (McPs) profile of poxviruses, table S1–S3

	Vaccinia virus WR	Variola virus DNA	Monkeypox virus strain Zaire-96-I-16	Yaba monkey tumor virus	Fowlpox virus	Canarypox virus	Orf virus	Cowpox virus	Camelpox virus	Myxoma virus strain Lausanne	Nile crocodilepox virus
GenBank ID	AY243312	X69198	AF380138	AY386371	AF198100	AY318871	AY386264	AF482758	AF438165	AF170726	DQ356948
Number of proteins	218	197	191	140	260	328	130	233	211	170	173
Protein interaction, thiol-disulfide transfer [25]											
CxxxC	25	23	22	16	27	41	15	32	28	16	36
CxxC	35	33	32	27	48	64	30	44	35	31	28
Binding to integrins, RGD-related motifs (3–8% of whole proteome) [26]											
RGD	9	6	10	5	8	10	11	7	10	6	14
%	4.1	3	5.2	3.6	3.1	3	8.5	3	4.7	3.5	8.1
Binding to phospholipids, lipid raft-mediated endocytosis (3–27% of proteome) [27]											
RxLR	12	8	10	6	12	19	36	14	11	5	38
%	5.5	4.1	5.2	4.3	4.6	5.8	27.7	6	5.2	2.9	22
Glycosylation sites (58–81% of proteome) - (http://prosite.expasy.org/PDOC00001)*											
N{P}[ST]{P}	165	153	154	112	209	264	78	181	167	128	101
%	75.7	77.7	80.6	80	80.4	80.5	60	77.7	79.1	75.3	58.4
Nuclear localization sequence (NLS; KR-rich) motifs [28]											
KRxR	11	10	10	6	8	18	9	17	13	10	19
KRx [10, 12] K[KR][KR]	0	0	0	0	1	4	1	0	0	0	1
KRx [10, 12] K[KR]X[KR]	1	1	2	2	1	5	0	2	0	0	2
K[KR]RK	3	3	2	2	6	8	0	3	3	2	5
KR[KR]R	1	1	1	1	0	3	2	1	2	1	7
[PR]xxKR{DE}[KR]	0	0	0	3	5	5	1	0	0	3	1
[RP]xxKR[KR]{DE}	1	2	0	2	4	2	3	2	1	2	2
RKRP	1	1	1	0	2	0	0	1	0	0	0
Protein folding, Rossmann folds motifs, bind FAD or NAD(P) [29]											
Gx [1, 2] GxxG	8	10	13	8	14	12	8	15	13	11	21
Gxxx[GA]	110	96	101	54	108	146	106	116	99	94	128
SUMO binding (40–58 and 40–61% of proteome) [12]											
[VI]x[VI][VI]	105	102	98	78	141	191	53	122	107	78	72
%	48.2	51.8	51.3	55.7	54.2	58.2	40.8	52.4	50.7	45.9	41.6
hKx[DE]	119	110	112	82	147	194	52	128	116	104	74
%	54.6	55.8	58.6	58.6	56.5	59.1	40	54.9	55	61.2	42.8
Recruit ESCRT pathway [30]											
YxxL	129	120	128	90	162	222	61	149	133	119	111
%	59.2	60.9	67	64.3	62.3	67.7	46.9	63.9	63	70	64.2
hPxV	42	41	42	30	53	79	44	47	41	51	72
%	19.3	20.8	22	21.4	20.4	24.1	33.8	20.2	19.4	30	41.6
Walker A, A’ and B motifs [31]											
[AG]xxxxGK[ST]	5	5	4	7	12	13	5	6	5	6	5
hhhhDxDxR	3	3	3	1	2	2	1	3	3	2	5
hhhDxxP	15	13	19	8	19	13	23	18	17	15	31

Total number of McPs (proteins harboring at least one instance of query motif; if >1 instances, they considered as (1) are counted for each query motifs; “%” means percentage of proteins (McPs) to total number of proteins; “x” denotes any residue; “{P}” denotes any residues, but P; alternative residues are bracketed; and [1, 2] means the motif is flanked by one or two residue(s); “h” denotes non-polar or hydrophobic residues. In this study, we considered h is equivalent “A, C, F, G, V, L, I, P, W, M, or Y” residue, Table S1

* Glycosylation sites were searched in entire protein sequences, but not confined to N- or C-terminals

Third module can parse UniProt and PROSITE flat files and convert them to human-readable tables, Figs. S7–S9. Shetti-Motif maps them into one table, which includes PROSITE IDs, patterns, and name of proteins harboring these patterns, Fig. S9. The tables can be copied into clipboard or can be exported into a tabulated text file.

### Implementation

Shetti-Motif tool, sample files, and documentation are available on http://sourceforge.net/projects/ShettiMotif/. The tool runs and it was tested on windows 7 or higher, without any preliminary installation. For Mac and Linux, MonoDevelop (http://www.monodevelop.com/) are needed. For details, see program’s user guide.

### Case study

**Fig. 2:**
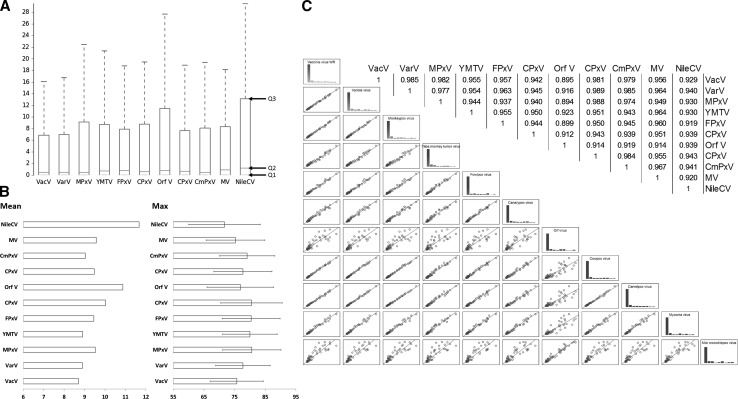
Poxviruses encode divergent number of motifs; the motif-containing proteins (McPs) profile of closely related viruses are correlated. The number of motif-containing proteins (i.e., protein containing at least one instance of the query motif) were counted and normalized (percentage) to total number of proteins encoded by a virus, Table 1, S3. **a** Box and whisker plot shows 1st, 2nd, 3rd quantiles (Q1, Q2 and Q3 respectively) of numbers of McPs, whiskers at 1.5 IQR (interquartile range) (Q3 + 1.5 IQR); **b** the average and maximum numbers of McPs, the *error bars* are based on standard deviation values; **c** Spearman correlation coefficient values and scatterplots (using STASTICA Data Miner; StatSoft, USA) of the number of McPs encoded by each virus. VacV: Vaccinia virus WR, VarV: Variola virus, MPxV: Monkeypox virus, YMTV: Yaba monkey tumor virus, FPxV: Fowlpox virus, CPxV: Canarypox virus, Orf V: Orf virus, CPxV: Cowpox virus, CmPxV: Camelpox virus M-96, MV: Myxoma virus, and NileCV: Nile crocodilepox virus

As a proof of concept, we analyzed proteomes encoded by eleven members of *Poxviridae* family (2251 proteins) against experimentally validated built-in motifs (Walker motifs, glycosylation, nuclear localization, SUMO-, ESCRT- and integrin-binding motifs, etc.), Tables 1, S2, S3 [1]. The viruses belong to *Chordopoxvirinae* (*Orthopoxvirus*: camelpox, cowpox, monkeypox, vaccinia and variola viruses; *Avipoxvirus*: canarypox and fowlpox viruses; *Crocodylidpoxvirus*: Nile crocodilepox virus; *Leporipoxvirus*: myxoma virus; *Parapoxvirus*: orf virus; and *Yatapoxvirus*: Yaba monkey tumor virus). Poxviruses are ubiquitous and infect wide-range of hosts [19]. Therefore, (i) entry, virus-cell interactions, or cellular trafficking mechanisms might not be conserved between species or subfamily members, and (ii) comparative proteomics approach is a potential benchmark to understand these interactions. First, the proteomes were searched for ≈100 query motifs, see Table 1, S2, S3. Then for each virus, the proteins harboring these motifs were counted and normalized to the total number of proteins in the proteome. Finally, the motif-profile table was constructed, Table 1, S3. For statistical analysis, the mean and maximum number of motif-containing proteins, standard deviation, and Spearman correlation coefficient were calculated, see Figs. 1, 2.

The results show that (i) the number of protein harboring these motifs significantly differs among poxviruses, Tables 1, S3, Fig. 2. Although a proteome harbors several copies of a motif, another proteome does not harbor any copy of the same motif (e.g.,. NLS motifs). (ii) The closely related viruses show a linear correlation, e.g., vaccinia and variola viruses (infect human cells and phylogenetically related) show similar motif-profile and Spearman correlation ≈0.99, Fig. 2. (iii) Some motifs were not detected in any of poxvirus proteomes (e.g., inhibitor of apoptosis, adenovirus fiber flexibility, and protein cleavage motifs, which characterize other viral families). This suggests that poxviruses encode wide range of proteins and functional motifs for fruitful interactions with wide range of host cells, and evolutionary events play roles to shape their proteome diversity. This explains the ubiquitous nature and ability of poxviruses to interact with wide range of hosts.

### Results and discussion

Shetti-Motif has a user-friendly interface in which plain data are visualized as a table, and can be copied to clipboard and transferred into spreadsheet program. The sequences containing the x-rich motifs are exported directly to a FASTA file. Thus, the input and output files can be managed easily by experimental biologists. Shetti-Motif searches for multiple pre-defined motifs/patterns within proteome or large dataset of protein sequences. This functionality does not require to searching public databases, loading a background sequence file, or writing additional scripts. This offers flexible option for biologists to search wide range of protein sequences, which are not indexed in public databases. This issue could be critical when parsing proteome datasets of recently isolated microbiological and metagenomics samples. To the best our knowledge, this whole-proteome mining approach cannot be achieved by similar tools. Shetti-Motif was used to search for ≈100 experimentally validated patterns against poxvirus proteomes. The results show variation in enrichment of motif-containing proteins among the viruses, which support that motifs are correlated with evolutionary events, cellular interaction, or host-specificity.

LCRs are sequence repeats or extension of one or more residue(s), e.g., 6xHis-tag. Despite their functional importance, they are under-represented on publications, reviewed in [1, 17, 20–22]. Their crystallization could be difficult; thus, previous efforts attempted to mask them. Another type of motifs, which are enriched with a residue(s) but interrupted by others, e.g., Cys-rich, Gly-rich or KR-rich motifs, reviewed in [1]. Notably, in literature, they are referred as x-rich motif, but not as LCRs. This could be due to the following: (i) they may not be considered as disordered repeats, (ii) may not conform to a known pattern, and (iii) could be structurally important. The difference between LCRs and x-rich motifs can be noticed in some proteins (e.g., Q5UNS9, E3VZK9, Q5UNX5, and Q5UQQ7), see SI-1, SI-2. Q5UNS9 harbors glycosylation sites LCRs, whereas the x-rich regions in the others are not masked by NCBI-BLASTp. For this reason, the criterion for finding x-rich motifs in Shetti-Motif is the coverage of the residue(s) to the total motif length. The x-rich proteins may share common biochemical or molecular interactions, e.g., post-translational modification for non-histone proteins. Therefore, it is beneficial to establish a dataset of proteins rich with particular residues, for investigating (experimentally) their molecular functions.

Short motifs are subjected to evolutionary changes, which could affect cellular processes, interactions, or protein characteristics [1–3]. Although proteins sharing functional motifs might share similar function, the consensus pattern is not the absolute measure for the protein functions, and other factors could influence the function, reviewed in [1]. Our bioinformatics approach may benefit in predicting tropism and pathogenicity for emerging infectious agents [23, 24], as well as determining potential protein dataset(s) among whole proteome for designing further experiments. Importantly, this approach includes exact text search of experimentally validated motifs, which increase the chances of true-positive results. However, motif-containing proteins may still have different functions from that being expected, which benefits studies on evolution of protein function.

*In conclusion*, Shetti-Motif has simple, versatile, user-friendly, and interactive features, which are useful for experimental biologists lacking prior knowledge of bioinformatics, such as search for pattern(s) or x-rich motifs in protein sequence(s) or entire proteome without loading background files and user-friendly interface to visualize UniProt and PROSITE flat files as tables.

We applied this pipeline to poxvirus proteomes, and we observed that our pipeline is able to correlate the closely related viruses. The results show that functional motifs are conserved within evolutionary related viruses and/or viruses that share similar molecular interactions. Therefore, we conclude that the pipeline is useful to compare between species; it will help in designing a dataset of candidate proteins for further experimental investigations, either by confirming the function or studying the evolution of protein function.

## Electronic supplementary material

Below is the link to the electronic supplementary material.

Figures S1–S9 (PDF 870 kb)

Table S2 (XLS 52 kb)

Table S3 (XLSX 25 kb)

Supplementary material 4 (TXT 939 kb)

Supplementary material 5 (TXT 12 kb)

Supplementary material 6 (TXT 14 kb)

Supplementary material 7 (TXT 446 kb)
