# Supplementary material for: A bioinformatics pipeline to search functional motifs within whole-proteome data: a case study of poxviruses
Source: Virus Genes. 2016 Dec 20;53(2):173–8. doi: 10.1007/s11262-016-1416-9 (PMC5357487; doi:10.1007/s11262-016-1416-9)
Supplement: Supplementary file 1 — Figures S1–S9 (PDF 870 kb) [file 11262_2016_1416_MOESM1_ESM.pdf]

Shetti-Motif

Haitham Sobhy

Supplementary file – **SI-1**: This file, contains table S1 and figures S1-S9.

Supplementary file – SI-2: A compressed file (zip format) contains NCBI-BLASTp output files. The x-rich motifs differ from LCRs and may not be masked. For example, BLASTp was used to mask LCRs in four proteins encoded by *Acanthamoeba polyphaga* mimivirus (UniProt ID: 5757), as the following:

1. Collagen-like protein (L669: Q5UNS9), 1937 aa, is S-rich protein (450 Ser, ~23% of length), the LCR regions are masked by BLASTp.
2. Hypothetical protein (R661: E3VZK9), 218 aa, is enriched with Cys (~11% of length), but the region masked does not contain Cys residue.
3. Hypothetical protein (L725: Q5UNX5), 224 aa, is enriched with Cys and Lys (~17% of the length; 21 K and 18 C), which are not masked.
4. Putative TPR repeat-containing protein (R856: Q5UQQ7), 342 aa, KLN share ~27% of R856 length, (32, 30 and 30 Asn, Lys and Leu, respectively, but only 5 Cys and 1 Pro), but not masked.

Supplementary Table S2 (in a separate excel spreadsheet).

The tool is supplemented with built-in patterns/motifs obtained from experimentally validated literature. The table consists of the motif, description/function and references to PubMed IDs. See also, Fig. S3.

Supplementary Table S3 (in a separate excel spreadsheet).

The motif-containing proteins were counted and normalized (percentage %) to the total number of the proteins encoded by a virus. These normalized values were then used for further statistical analysis, see Fig. 2 in the manuscript.

Supplementary file – S1

Table S1. Shetti-Motif accepts PROSITE pattern syntax. Some reports use Latin symbols to refer to degenerate residues, e.g. θ, Ψ, Φ, etc., which refers to residues share a similar physicochemical property(ies), such as hydrophobicity, polarity, etc. For simplicity, Shetti-Motif accepts non-Latin symbols as listed in the table. These symbols are explicitly translated to the corresponding residues. Otherwise, users may insert alternative / degenerate residues between "[" and "]".

Noteworthy, the physical properties of bases might be not the same within literatures. As an example, some reports refer to (F, I, L or V) residues as hydrophobic one. In Shetti-Motif, A, C, F, G, V, L, I, P, W, M or Y are considered as hydrophobic residues. This may lead to reporting additional motifs (e.g. contain A or Y, instead of those contain only F, I, L or V). To overcome this issue, users may write directly "[FILV]", instead of the representative symbol.

Table S1.

**Residues**	**Representative Motif**	**Symbol**	**Physical properties**
Arg-Gly-Asp	RGD		
Pro-Pro-any amino acid-Tyr	PPxY		
P, and (E or D) residues ≈ PE or PD	P[ED]		
P, and any residue but R	P{R}		
T is repeated 2, 2-5 or >3 times	PAST(2), PAST(2-5), or PAST(3,)		
E or D	[ED]	- "hyphen"	Negative - Acidic
H, K or R	[HKR]	+	Positive - Basic
S or T	[ST]	=	Alcohol
C or M	[CM]	*	Sulfur containing
I, V or L	[IVL]	?	Aliphatic
A, G or S	[AGS]	&	Tiny
F, H, W or Y	[FHWY]	@	Aromatic
D, E, H, K, N, Q, R, S or T	[DEHKNQRST]	%	Polar / hydrophilic
A, C, F, G, V, L, I, P, W, M or Y	[ACFGVLIPWMY]	!	Non-polar / hydrophobic
C, W, N, Q, S, T, Y, K, R, H, D or E	[CWNQSTYKRHDE]	#	H-bond
Any amino acid	Any amino acid	x	Any amino acid

Fig. S1. Result of sequences enriched with residues (x-rich motifs) appears in "Result - statistics" tab, which include the names of the proteins, protein length, number of motifs in each protein, and coverage of the residues to the full length of the protein (%). The results are tabulated and can be transferred to text editor or Excel software.

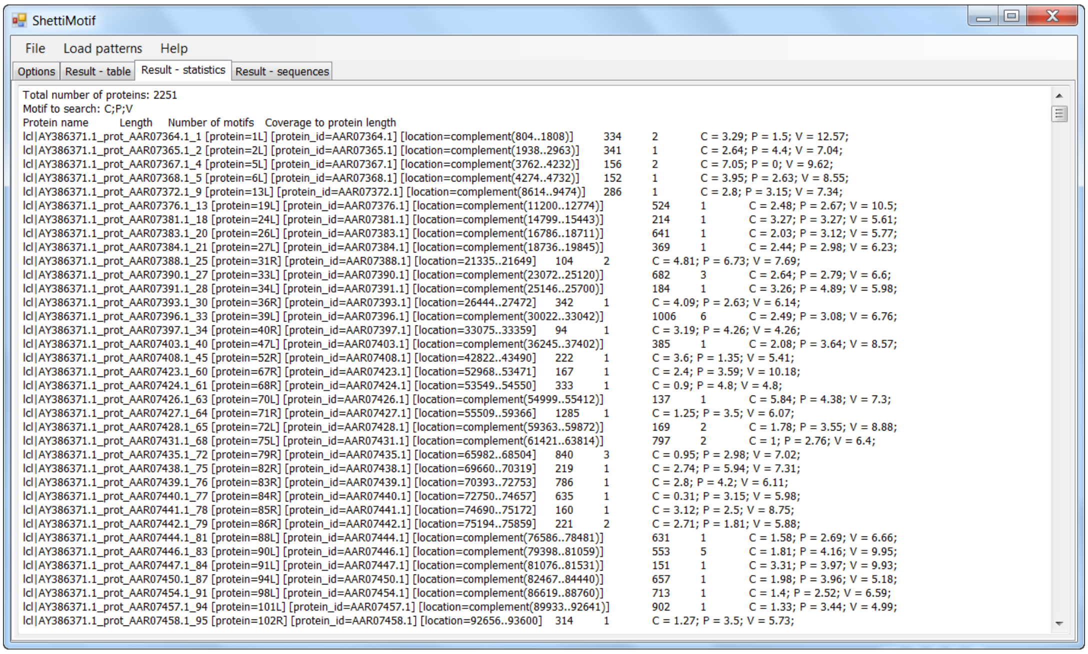

Fig. S2. For sequences enriched with residues (x-rich motifs), the protein sequence headers, the sequences of the motifs and statistics are presented in tab “Result - sequences”. The results can be transferred to text editor software.

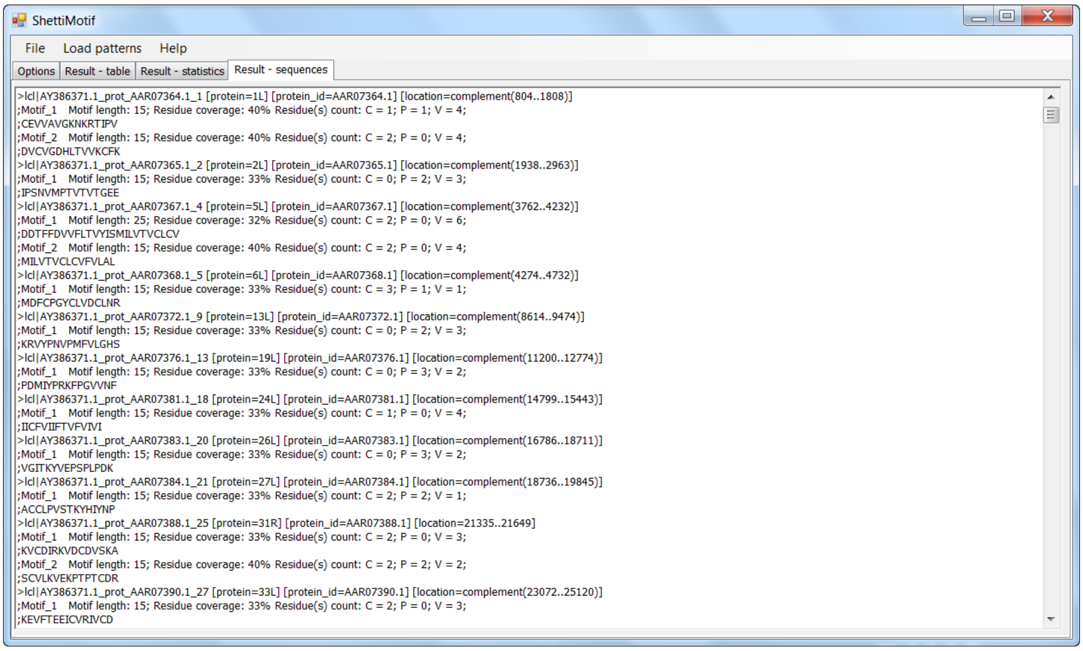

Fig. S3. The built-in patterns are listed with check-boxes. The user may choose the pattern(s) of interest, then click on “Select these patterns” button. The pattern(s) are populated in the search text area. In “Options” tab, select or deselect all by ticking on “Select All”. The patterns were acquired from PROSITE databases (http://prosite.expasy.org/, ftp://ftp.expasy.org/databases/prosite/, last accessed August, 2015), or from literatures, see table S2 for references and PubMed IDs.

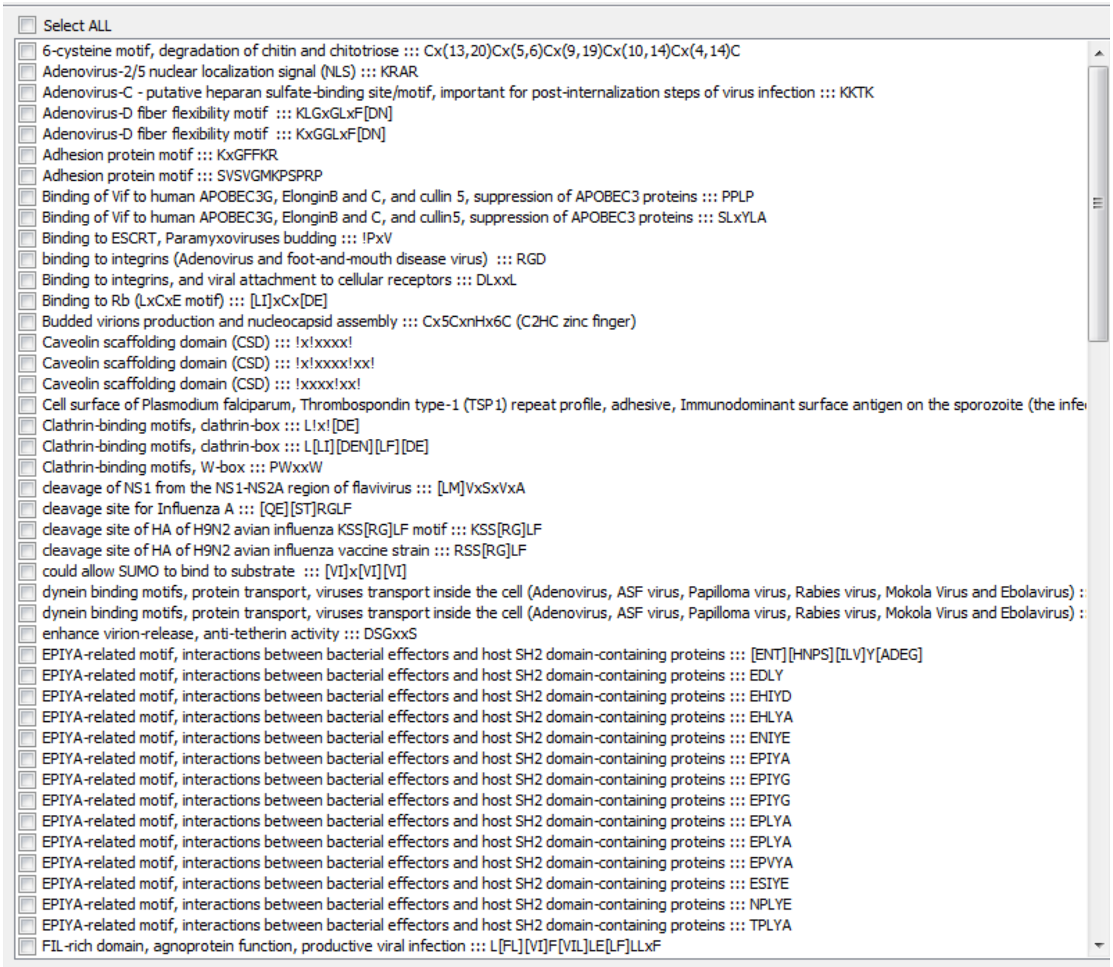

Fig. S4. After clicking “Search pattern” button, the protein containing the motifs, and the motifs and their positions on the sequences are listed in a table, in “Result - table” tab. The results are tabulated and can be moved to text editor or Excel software.

Note: for multiple pattern, the results are printed to text file.

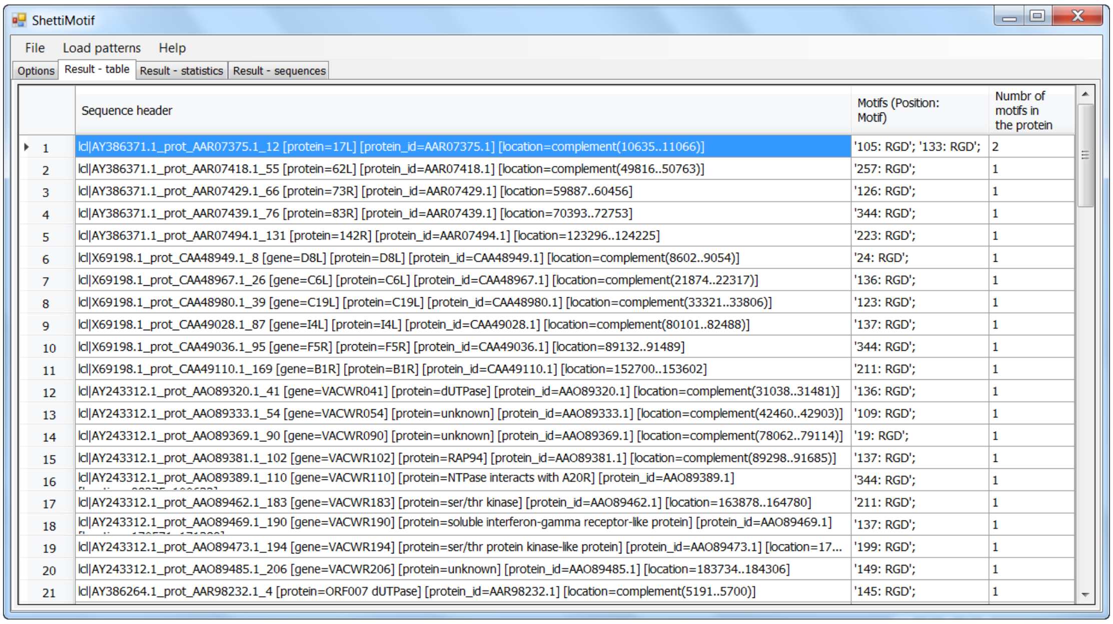

	Sequence header	Motifs (Position: Motif)	Numbr of motifs in the protein
1	lcl|AY386371.1_prot_AAR07375.1_12 [protein=17L] [protein_id=AAR07375.1] [location=complement(10635..11066)]	'105: RGD'; '133: RGD';	2
2	lcl|AY386371.1_prot_AAR07418.1_55 [protein=62L] [protein_id=AAR07418.1] [location=complement(49816..50763)]	'257: RGD';	1
3	lcl|AY386371.1_prot_AAR07429.1_66 [protein=73R] [protein_id=AAR07429.1] [location=59887..60456]	'126: RGD';	1
4	lcl|AY386371.1_prot_AAR07439.1_76 [protein=83R] [protein_id=AAR07439.1] [location=70393..72753]	'344: RGD';	1
5	lcl|AY386371.1_prot_AAR07494.1_131 [protein=142R] [protein_id=AAR07494.1] [location=123296..124225]	'223: RGD';	1
6	lcl|X69198.1_prot_CAA48949.1_8 [gene=D8L] [protein=D8L] [protein_id=CAA48949.1] [location=complement(8602..9054)]	'24: RGD';	1
7	lcl|X69198.1_prot_CAA48967.1_26 [gene=C6L] [protein=C6L] [protein_id=CAA48967.1] [location=complement(21874..22317)]	'136: RGD';	1
8	lcl|X69198.1_prot_CAA48980.1_39 [gene=C19L] [protein=C19L] [protein_id=CAA48980.1] [location=complement(33321..33806)]	'123: RGD';	1
9	lcl|X69198.1_prot_CAA49028.1_87 [gene=I4L] [protein=I4L] [protein_id=CAA49028.1] [location=complement(80101..82488)]	'137: RGD';	1
10	lcl|X69198.1_prot_CAA49036.1_95 [gene=F5R] [protein=F5R] [protein_id=CAA49036.1] [location=89132..91489]	'344: RGD';	1
11	lcl|X69198.1_prot_CAA49110.1_169 [gene=B1R] [protein=B1R] [protein_id=CAA49110.1] [location=152700..153602]	'211: RGD';	1
12	lcl|AY243312.1_prot_AAO89320.1_41 [gene=VACWR041] [protein=dUTPase] [protein_id=AAO89320.1] [location=complement(31038..31481)]	'136: RGD';	1
13	lcl|AY243312.1_prot_AAO89333.1_54 [gene=VACWR054] [protein=unknown] [protein_id=AAO89333.1] [location=complement(42460..42903)]	'109: RGD';	1
14	lcl|AY243312.1_prot_AAO89369.1_90 [gene=VACWR090] [protein=unknown] [protein_id=AAO89369.1] [location=complement(78062..79114)]	'19: RGD';	1
15	lcl|AY243312.1_prot_AAO89381.1_102 [gene=VACWR102] [protein=RAP94] [protein_id=AAO89381.1] [location=complement(89298..91685)]	'137: RGD';	1
16	lcl|AY243312.1_prot_AAO89389.1_110 [gene=VACWR110] [protein=NTPase interacts with A20R] [protein_id=AAO89389.1] [illegible]	'344: RGD';	1
17	lcl|AY243312.1_prot_AAO89462.1_183 [gene=VACWR183] [protein=ser/thr kinase] [protein_id=AAO89462.1] [location=163878..164780]	'211: RGD';	1
18	lcl|AY243312.1_prot_AAO89469.1_190 [gene=VACWR190] [protein=soluble interferon-gamma receptor-like protein] [protein_id=AAO89469.1] [illegible]	'137: RGD';	1
19	lcl|AY243312.1_prot_AAO89473.1_194 [gene=VACWR194] [protein=ser/thr protein kinase-like protein] [protein_id=AAO89473.1] [location=17...	'199: RGD';	1
20	lcl|AY243312.1_prot_AAO89485.1_206 [gene=VACWR206] [protein=unknown] [protein_id=AAO89485.1] [location=183734..184306]	'149: RGD';	1
21	lcl|AY386264.1_prot_AAR98232.1_4 [protein=ORF007 dUTPase] [protein_id=AAR98232.1] [location=complement(5191..5700)]	'145: RGD';	1

Fig. S5. The percentage (in %) of the protein harbouring these motifs to the entire protein dataset (proteome) appears in a text area, in "Result - statistics" tab. The results are tabulated and can be moved to text editor or Excel software.

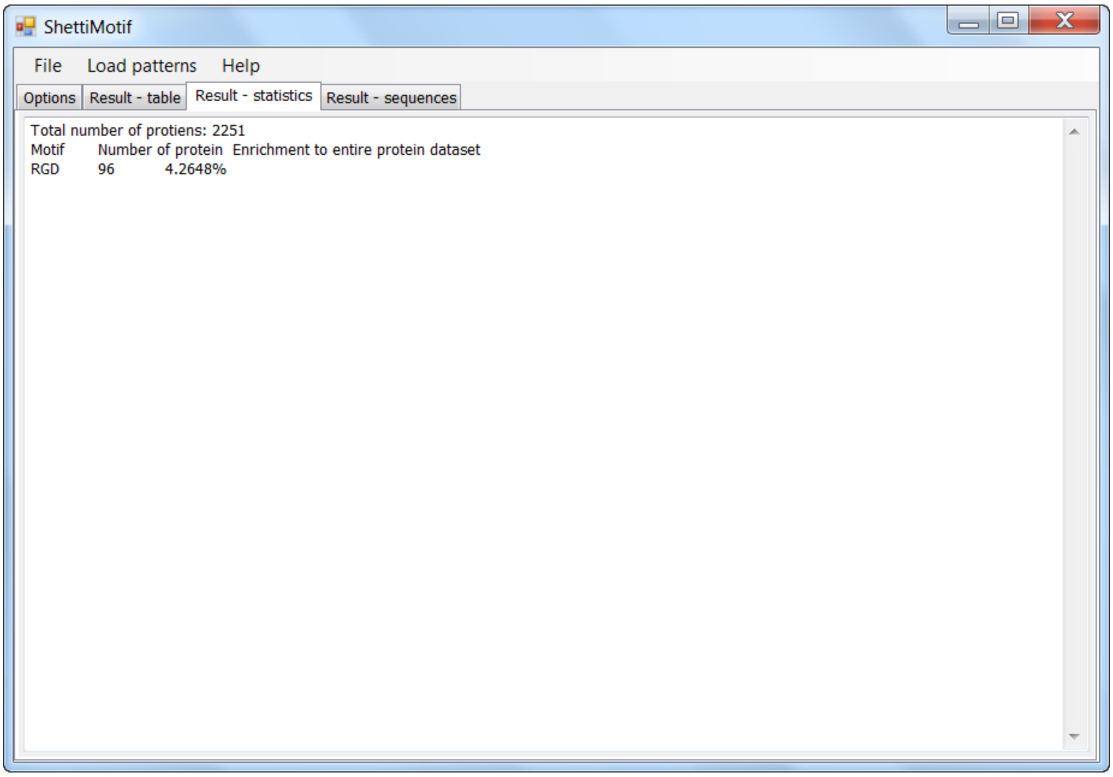

Fig. S6. The sequences of the motif-containing proteins appear in a text area, in “Result - sequences” tab, which can be copied to text editor software.

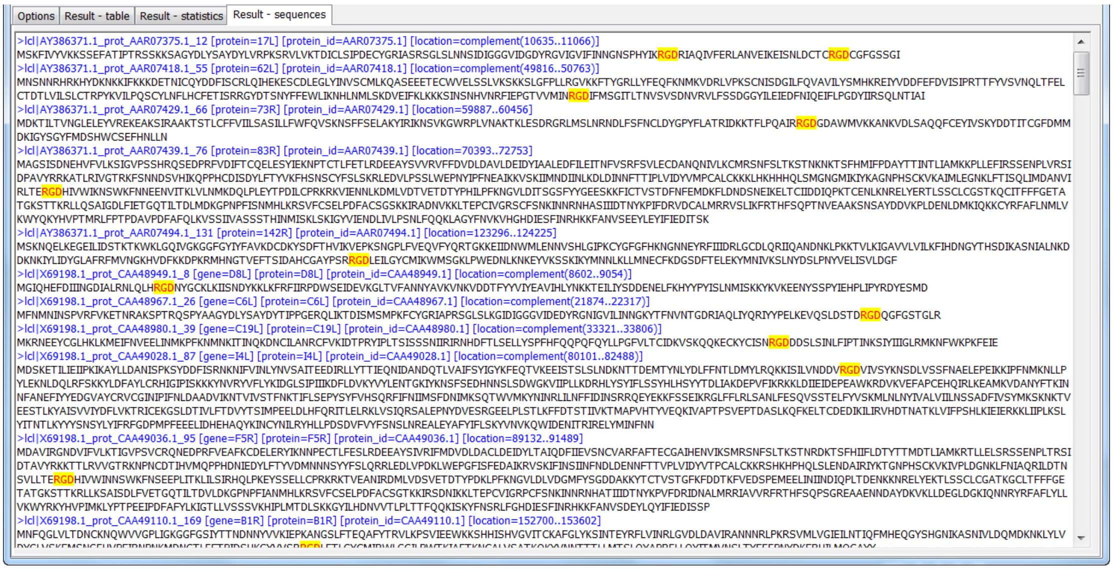

Fig. S7. The PROSITE flat file can be converted to a table format. In “Result - table” tab, the results are tabulated and can be moved to text editor or Excel software.

	Identification	Accession number	Description	Pattern
1	ASN_GLYCOSYLATION; PATTERN	PS00001	N-glycosylation site	N-{P}-[ST]-{P}
2	CAMP_PHOSPHO_SITE; PATTERN	PS00004	cAMP- and cGMP-dependent protein kinase phosphorylation site	[RK](2)-x-[ST]
3	PKC_PHOSPHO_SITE; PATTERN	PS00005	Protein kinase C phosphorylation site	[ST]-x-[RK]
4	CK2_PHOSPHO_SITE; PATTERN	PS00006	Casein kinase II phosphorylation site	[ST]-x(2)-[DE]
5	TYR_PHOSPHO_SITE; PATTERN	PS00007	Tyrosine kinase phosphorylation site	[RK]-x(2,3)-[DE]-x(2,3)-Y
6	MYRISTYL; PATTERN	PS00008	N-myristoylation site	G-{EDRKHPFYW}-x(2)-[STAGCN]-{P}
7	AMIDATION; PATTERN	PS00009	Amidation site	x-G-[RK]-[RK]
8	ASX_HYDROXYL; PATTERN	PS00010	Aspartic acid and asparagine hydroxylation site	C-x-[DN]-x(4)-[FY]-x-C-x-C
9	GLA_1; PATTERN	PS00011	Vitamin K-dependent carboxylation domain	E-x(2)-[ERK]-E-x-C-x(6)-[EDR]-x(10,11)-[FYA]-[YW]
10	PHOSPHOPANTETHEINE; PATTERN	PS00012	Phosphopantetheine attachment site	[DEQGSTALMKRH]-[LIVMFYSTAC]-[GNQ]-[LIVMFYAG]-[DNEKHS]-S-[LIVMST]-{PCF
11	ER_TARGET; PATTERN	PS00014	Endoplasmic reticulum targeting sequence	[KRHQSA]-[DENQ]-E-L>
12	RGD; PATTERN	PS00016	Cell attachment sequence	R-G-D
13	ATP_GTP_A; PATTERN	PS00017	ATP/GTP-binding site motif A (P-loop)	[AG]-x(4)-G-K-[ST]
14	EF_HAND_1; PATTERN	PS00018	EF-hand calcium-binding domain	D-{W}-[DNS]-{ILVFYW}-[DENSTG]-[DNQGHRK]-{GP}-[LIVMC]-[DENQSTAGC]-x(2)
15	ACTININ_1; PATTERN	PS00019	Actinin-type actin-binding domain signature 1	[EQ]-{LNYH}-x-[ATV]-[FY]-{LDAM}-{T}-W-{PG}-N
16	ACTININ_2; PATTERN	PS00020	Actinin-type actin-binding domain signature 2	[LIVM]-x-[SGNL]-[LIVMN]-[DAGHENRS]-[SAGPNVT]-x-[DNEAG]-[LIVM]-x-[DEAGQ]-
17	KRINGLE_1; PATTERN	PS00021	Kringle domain signature	[FY]-C-[RH]-[NS]-x(7,8)-[WY]-C
18	EGF_1; PATTERN	PS00022	EGF-like domain signature 1	C-x-C-x(2)-{V}-x(2)-G-{C}-x-C
19	FN2_1; PATTERN	PS00023	Fibronectin type-II collagen-binding domain signature	C-x(2)-P-F-x-[FYWIV]-x(7)-C-x(8,10)-W-C-x(4)-[DNSR]-[FYW]-x(3,5)-[FYW]-x-[F
20	HEMOPEXIN; PATTERN	PS00024	Hemopexin domain signature	[LIFAT]-{IL}-x(2)-W-x(2,3)-[PE]-x-{VF}-[LIVMFY]-[DENQS]-[STA]-[AV]-[LIVMFY]
21	P_TREFOIL_1; PATTERN	PS00025	P-type 'Trefoil' domain signature	[KRH]-x(2)-C-x-[FYPSTV]-x(3,4)-[ST]-x(3)-C-x(4)-C-C-[FYWH]
22	CHIT_BIND_I_1; PATTERN	PS00026	Chitin recognition or binding domain signature	C-x(4,5)-C-C-S-x(2)-G-x-C-G-x(3,4)-[FYW]-C
23	HOMEOBOX_1; PATTERN	PS00027	'Homeobox' domain signature	[LIVMFYG]-[ASLVR]-x(2)-[LIVMSTACN]-x-[LIVM]-{Y}-x(2)-{L}-[LIV]-[RKNQESTAIY]
24	ZINC_FINGER_C2H2_1; PATTERN	PS00028	Zinc finger C2H2 type domain signature	C-x(2,4)-C-x(3)-[LIVMFYWC]-x(8)-H-x(3,5)-H
25	LEUCINE_ZIPPER; PATTERN	PS00029	Leucine zipper pattern	L-x(6)-L-x(6)-L-x(6)-L
26	NUCLEAR_REC_DBD_1; PATTERN	PS00031	Nuclear hormones receptors DNA-binding region signature	C-x(2)-C-x(1,2)-[DENAVSPHKQT]-x(5,6)-[HNY]-[FY]-x(4)-C-x(2)-C-x(2)-F(2)-x-R
27	ANTENNAPEDIA; PATTERN	PS00032	'Homeobox' antennapedia-type protein signature	[LIVMFE]-[FY]-P-W-M-[KRQTA]
28	ENGRAILED; PATTERN	PS00033	'Homeobox' engrailed-type protein signature	L-M-A-[EQ]-G-L-Y-N

Fig. S8. The UniProt flat file is converted to a table format. The table includes UniProt ID, name of organism, protein name, taxonomy, sequence, PROSITE pattern IDs, InterPro and Pfam IDs. The results are tabulated, and blank cells denotes absence of those IDs.

	Identification	Accession number	Description (recommended name)	Gene name	Organism species	Organelle	Length	MW	PROSITE	InterPro	Pfam
21	DHCR7_MIMIV	Q5UQI4	Probable 7-dehydroc…	MIMI_R807	Acanthamoeba polyphaga mimivirus (APMV)		447	51692		IPR001171;	PF01222;
22	DIPP_MIMIV	Q5UQW2	Putative	MIMI_L375	Acanthamoeba polyphaga mimivirus (APMV)		360	43662	PS51462;PS50158;	IPR000086;IPR0157	PF00293;
23	DNLJ_MIMIV	Q5UPZ0	DNA ligase	MIMI_R303	Acanthamoeba polyphaga mimivirus (APMV)		636	72089	PS50172;	IPR001357;IPR0…	PF00533;PF016…
24	DNMK_MIMIV	Q5UQ70	Putative	MIMI_R512	Acanthamoeba polyphaga mimivirus (APMV)		193	22106		IPR027417;	
25	DPOLX_MIMIV	Q7T6Y4	Probable DNA polyme…	MIMI_L318	Acanthamoeba polyphaga mimivirus (APMV)		354	40612	PS00522;	IPR002054;IPR0…	PF14792;PF147…
26	DPOL_MIMIV	Q5UQR0	DNA polymerase	MIMI_R322	Acanthamoeba polyphaga mimivirus (APMV)		1740	201945	PS50818;PS50819;	IPR006172;IPR0061	PF00136;PF03104
27	DRTS_MIMIV	Q5UQG3	Bifunctional dihydrofo…	MIMI_R497	Acanthamoeba polyphaga mimivirus (APMV)		563	65063	PS51330;PS00091;	IPR024072;IPR0…	PF00186;PF00303
28	END4_MIMIV	Q5UPY4	Putative endonuclease 4	MIMI_R296	Acanthamoeba polyphaga mimivirus (APMV)		312	34857	PS00730;PS51432;	IPR001719;IPR0182	PF01261;
29	FPG_MIMIV	Q5UQ00	Probable formamidop…	MIMI_L315	Acanthamoeba polyphaga mimivirus (APMV)		287	33463	PS51068;	IPR015886;IPR0…	PF01149;PF06831
30	GFAT_MIMIV	Q7T6X6	Probable glutamine--fructose-6-ph aminotransferase	MIMI_L619	Acanthamoeba polyphaga mimivirus (APMV)		606	68570	PS51278;PS51464;	IPR017932;IPR0058	PF01380;
31	GLNA_MIMIV	Q5UR44	Putative glutamine sy…	MIMI_R565	Acanthamoeba polyphaga mimivirus (APMV)		353	40079	PS00180;PS00181;	IPR008147;IPR0…	PF00120;PF03951
32	GLRX_MIMIV	Q5UQ14	Probable glutaredoxin	MIMI_R195	Acanthamoeba polyphaga mimivirus (APMV)		106	12084	PS00195;PS51354;	IPR011767;IPR0021	PF00462;
33	GNA1_MIMIV	Q5UPZ9	Probable glucosamine…	MIMI_L316	Acanthamoeba polyphaga mimivirus (APMV)		148	17017	PS51186;	IPR016181;IPR0…	PF00583;
34	HSP70_MIMIV	Q5UQ49	Heat shock 70 kDa protein homolog	MIMI_L393	Acanthamoeba polyphaga mimivirus (APMV)		634	70514	PS00297;PS00329;PS01	IPR018181;IPR0290	PF00012;
35	HSP7L_MIMIV	Q5UPU0	Heat shock protein 7…	MIMI_L254	Acanthamoeba polyphaga mimivirus (APMV)		941	107204	PS01036;	IPR018181;IPR0…	PF00012;
36	IF4EH_MIMIV	Q5UQG4	Eukaryotic translation initiation factor 4E homolog	MIMI_L496	Acanthamoeba polyphaga mimivirus (APMV)		272	30949		IPR023398;IPR0010	PF01652;
37	KITH_MIMIV	Q5UP25	Thymidine kinase	MIMI_L258	Acanthamoeba polyphaga mimivirus (APMV)		225	25909	PS00603;	IPR027417;IPR0…	PF00265;
38	LONH_MIMIV	Q5UPT0	Lon protease homolog	MIMI_L251	Acanthamoeba polyphaga mimivirus (APMV)		1023	116827		IPR003593;IPR0039	PF00004;PF05362
39	MCAR_MIMIV	Q5UPV8	Mitochondrial carrier-l…	MIMI_L276	Acanthamoeba polyphaga mimivirus (APMV)		237	27319	PS50920;	IPR018108;IPR0…	PF00153;
40	MCE_MIMIV	Q5UQX1	Probable mRNA-capping enzyme	MIMI_R382	Acanthamoeba polyphaga mimivirus (APMV)		1170	136508	PS51562;	IPR023577;IPR0013	PF01331;PF02940
41	MGMT_MIMIV	Q5UNU9	Probable methylated-…	MIMI_R693	Acanthamoeba polyphaga mimivirus (APMV)		149	16851	PS00374;	IPR001497;IPR0…	PF01035;
42	MUTSL_MIMIV	Q5UQU6	Putative DNA mismatch repair protein mutS homolog L359	MIMI_L359	Acanthamoeba polyphaga mimivirus (APMV)		1124	130330	PS00486;	IPR007695;IPR0004	PF01624;PF05192

Fig. S9. Combining (mapping) both UniProt and PROSITE files results in a new table, which includes PROSITE ID, accession numbers of the protein containing the pattern, description of the pattern and the pattern consensus. The results are tabulated, and blank cells denotes absence of consensus pattern in PROSITE flat file.

	PROSITE	Accession numbers	Description	Pattern
1	PS51278	Q5UQE1;Q7T6X6;	Glutamine amidotransferase type 2 domain profile	
2	PS00122	Q5UR02;	Carboxylesterases type-B serine active site	F[GR]Gx(4)[LIVM]x[LIV]xGxS[STAG]G
3	PS00941	Q5UR02;	Carboxylesterases type-B signature 2	[EDA][DG]CL[YTF][LIVT][DNS][LIV][LIVFYW]x[PQR]
4	PS00086	Q5UQI3;	Cytochrome P450 cysteine heme-iron ligand signature	[FW][SGNH]x[GD]{F}[RKHPT]{P}C[LIVMFAP][GAD]
5	PS00191	Q5UR80;	Cytochrome b5 family, heme-binding domain signature	[FY][LIVMK]{I}{Q}HP[GA]G
6	PS50255	Q5UR80;	Cytochrome b5 family, heme-binding domain profile	
7	PS51462	Q5UQW2;	Nudix hydrolase domain profile	
8	PS50158	Q5UQW2;	Zinc finger CCHC-type profile	
9	PS50172	Q5UPZ0;	BRCT domain profile	
10	PS00522	Q7T6Y4;	DNA polymerase family X signature	G[SG][LFY]xR[GE]x(3)[SGCL]xD[LIVM]D[LIVMFY](3)x(2)[SAP]
11	PS50818	Q5UQR0;	Intein C-terminal splicing motif profile	
12	PS50819	Q5UQR0;	Intein DOD-type homing endonuclease domain profile	
13	PS51330	Q5UQG3;	Dihydrofolate reductase (DHFR) domain profile	
14	PS00091	Q5UQG3;	Thymidylate synthase active site	Rx(2)[LIVMT]x(2,3)[FWY][QNYDI]x(8,13)[LVESI]xPC[HAVMLC]x(3)[QMTLHD][FYWL]x(0,1)[LV]
15	PS00730	Q5UPY4;	AP endonucleases family 2 signature 2	[GSARY][LIVMF][CT][LIVMFY]DTCH
16	PS51432	Q5UPY4;	AP endonucleases family 2 profile	
17	PS51068	Q5UQ00;	Formamidopyrimidine-DNA glycosylase catalytic domain profile	
18	PS51464	Q7T6X6;	SIS domain profile	
19	PS00180	Q5UR44;	Glutamine synthetase signature 1	[FYWL]DGSSx(6,8)[DENQSTAK][SA][DE]x(2)[LIVMFY]
20	PS00181	Q5UR44;	Glutamine synthetase putative ATP-binding region signature	KP[LIVMFYA]x(3,5)[NPAT][GA][GSTAN][GA]xHx(3)S
21	PS00195	Q5UQ14;	Glutaredoxin active site	[LIVMD][FYSA]x(4)C[PV][FYWH]Cx(2)[TAV]x(2,3)[LIV]
22	PS51354	Q5UQ14;	Glutaredoxin domain profile	
23	PS51186	Q5UPZ9;Q5UR52;	Gcn5-related N-acetyltransferase (GNAT) domain profile	
24	PS00297	Q5UQ49;	Heat shock hsp70 proteins family signature 1	[IV]DLGT[ST]x[SC]
25	PS00329	Q5UQ49;	Heat shock hsp70 proteins family signature 2	[LIVMF][LIVMFY][DN][LIVMFS]G[GSH][GS][AST]x(3)[ST][LIVM][LIVMFC]
26	PS01036	Q5UQ49;Q5UPU0;	Heat shock hsp70 proteins family signature 3	[LIVMY]x[LIVMF]xGGx[ST]{LS}[LIVM]Px[LIVM]x[DEQKRSTA]
27	PS00603	Q5UP25;	Thymidine kinase cellular-type signature	[GA]x(1,2)[DE]xYx[STAPV]xC[NKR]x[CH][LIVMFYWH]
28	PS50920	Q5UPV8;	Solute carrier (Solcar) repeat profile	
[illegible]	[illegible]	[illegible]	[illegible]	
